# Evaluating genome sequencing strategies: trio, singleton, and standard testing in rare disease diagnosis

**DOI:** 10.1186/s13073-025-01516-7

**Published:** 2025-09-18

**Authors:** Daniel Kaschta, Christina Post, Franziska Gaass, Milad Al-Tawil, Vincent Arriens, Saranya Balachandran, Tobias Bäumer, Valerie Berge, Friederike Birgel, Andreas Dalski, Maike Dittmar, Andre Franke, Sören Franzenburg, Janina Fuß, Bettina Gehring, Rebecca Gembicki, Bianca Greiten, Kristin Grohte, Britta Hanker, Kristian Händler, Lana Harder, Yorck Hellenbroich, Theresia Herget, Gloria Herrmann, Olaf Hiort, Kirstin Hoff, Birga Hoffmann, Nadine Hornig, Irina Hüning, Monika Kautza-Lucht, Juliane Köhler, Anna-Sophie Liegmann, Jasmin Lisfeld, Britt-Sabina Löscher, Nils G. Margraf, Michelle Meyenborg, Anna Möllring, Hiltrud Muhle, Eva Maria Murga Penas, Henning Nommels, Dzhoy Papingi, Imke Poggenburg, Jelena Pozojevic, Philip Rosenstiel, Andreas Recke, Kimberly Roberts, Laelia Rösler, Franka Rust, Maj-Britt Salewski, Katharina Schau-Römer, Christian Schlein, Varun K.A. Sreenivasan, Louiza Toutouna, Caroline Utermann-Thüsing, Amelie T. van der Ven, Alexander E. Volk, Janne Wehnert, Sandra Wilson, Rixa Woitschach, Veronica Yumiceba, Christine Zühlke, Alexander Münchau, Norbert Brüggemann, Inga Vater, Almuth Caliebe, Inga Nagel, Malte Spielmann

**Affiliations:** 1 Institute of Human Genetics, University Medical Center Schleswig-Holstein, University of Lübeck & Kiel University, Lübeck, Germany; 2 Department of Neonatology and Pediatric Intensive Care Medicine, Westphalian Children’s Center Dortmund, University of Witten-Herdecke, Witten, Germany; 3 Institute of Systems Motor Science, University Medical Center Schleswig-Holstein, University of Lübeck, Lübeck, Germany; 4 Institute of Medical Genetics, University of Zürich, Zurich, Switzerland; 5 Institute of Clinical Molecular Biology, Kiel University, Kiel, Germany; 6 Neurology Center, Itzehoe, Germany; 7 Institute of Tumorgenetic Nord, Kiel, Germany; 8 Institute of Human Genetics, University Medical Center Hamburg-Eppendorf, Hamburg, Germany; 9 Division of Paediatric Endocrinology and Diabetes, Department of Paediatrics and Adolescent Medicine, University Medical Center Ulm, Ulm, Germany; 10 Division of Paediatric Endocrinology and Diabetes, Department of Paediatrics and Adolescent Medicine, University of Lübeck, Lübeck, Germany; 11 Practice for Neuropaediatrics, Lüneburg, Germany; 12 Epilepsy Center Kiel, Department of Neurology, University Hospital Schleswig-Holstein, Christian-Albrechts-University, Kiel, Germany; 13 Department of Neurology, University Medical Center Schleswig-Holstein, University of Lübeck & Kiel University , Kiel, Germany; 14 Department of Neuropediatrics, University Medical Center Schleswig-Holstein, Christian-Albrechts University, Kiel, Germany; 15 Group Practice for Pediatrics and Adolescent Medicine, Husum, Germany; 16 Department of Dermatology, Allergology and Venereology, University Medical Center Schleswig-Holstein, University of Lübeck, Lübeck, Germany; 17 Center for Rare Diseases, University Medical Center Schleswig-Holstein, University of Kiel, Kiel, Germany; 18 Institute of Human Genetics, Faculty of Medicine, University Medical Center Freiburg, University of Freiburg, Freiburg, Germany; 19 Center for Rare Diseases, University Medical Center Schleswig-Holstein, University of Lübeck, Lübeck, Germany; 20 Section for Movement Disorder, Department of Neurology, University Medical Center Schleswig-Holstein, University of Lübeck, Lübeck, Germany; 21 Institute of Neurogenetics, University Medical Center Schleswig-Holstein, University of Lübeck, Lübeck, Germany; 22 DZHK (German Centre for Cardiovascular Research), Partner SiteHamburg/ , Lübeck/Kiel, Lübeck, Germany

**Keywords:** Genome sequencing, Exome sequencing, Standard of care, Rare disease, Diagnostic yield

## Abstract

**Background:**

Short-read genome sequencing (GS) is among the most comprehensive genetic testing methods available, capable of detecting single-nucleotide variants, copy-number variants, mitochondrial variants, repeat expansions, and structural variants in a single assay. Despite its technical advantages, the full clinical utility of GS in real-world diagnostic settings remains to be fully established.

**Methods:**

This study systematically compared singleton GS (sGS), trio GS (tGS), and exome sequencing-based standard-of-care (SoC) genetic testing in 416 patients with rare diseases in a blinded, prospective study. Three independent teams with divergent baseline expertise evaluated the diagnostic yield of GS as a unifying first-tier test and directly compared its variant detection capabilities, learning curve, and clinical feasibility. The SoC team had extensive prior experience in exome-based diagnostics, while the sGS and tGS teams were newly trained in GS interpretation. Diagnostic yield was assessed through both prospective and retrospective analyses.

**Results:**

In our prospective analysis, tGS achieved the highest diagnostic yield for likely pathogenic/pathogenic variants at 36.1% in the newly trained team, surpassing the experienced SoC team at 35.1% and the newly trained sGS team at 28.8%. To evaluate which variants could technically be identified and account for differences in team experience, we conducted a retrospective analysis, achieving diagnostic yields of 36.7% for SoC, 39.1% for sGS, and 40.0% for tGS. The superior yield of GS was attributed to its ability to detect deep intronic, non-coding, and small copy-number variants missed by SoC. Notably, tGS identified three de novo variants classified as likely pathogenic based on recent GeneMatcher collaborations and newly published gene-disease association studies.

**Conclusions:**

Our findings demonstrate that GS, particularly tGS, outperforms SoC in diagnosing rare diseases, with sGS providing a more cost-effective alternative. These results suggest that GS should be considered a first-tier genetic test, offering an efficient, single-step approach to reduce the diagnostic odyssey for patients with rare diseases. The trio approach proved especially valuable for less experienced teams, as inheritance data facilitated variant interpretation and maintained high diagnostic yield, while experienced teams achieved comparable results with singleton analysis alone.

**Supplementary Information:**

The online version contains supplementary material available at 10.1186/s13073-025-01516-7.

## Background

Advances in healthcare have steadily contributed to a global decline in disease burden [1]. With growing insights into the mechanisms and aetiology of diseases, recent studies reveal that genetic factors outweigh environmental contributions in approximately 40% of disease phenotypes [2]. Notably, in cases of infant mortality, genetic associations have been identified in 41% of instances, primarily linked to rare genetic variants [3]. Rare diseases, in general, represent a significant public health burden due to their complexity, limited treatment options, and high diagnostic and therapeutic costs [4]. In Germany, approximately four million people are affected [5]. In Western populations, between 6000 and 10,000 distinct rare diseases impact 3.5–5.9% of individuals, with over 80% believed to have a genetic origin [6, 7]. The combination of traditional diagnostic methods, including karyotyping, array comparative genomic hybridisation (array-CGH), and exome sequencing (ES), remains the standard of care (SoC) for establishing a broad range of molecular diagnoses. Although effective, when used together these methods are time-consuming and still face significant limitations in detecting non-coding variants, smaller structural variants, and complex genomic rearrangements due to inherent limitations in resolution [8–11]. Short-read genome sequencing (GS) presents a promising alternative to these traditional methods, offering a more comprehensive strategy [12]. Unlike targeted approaches, GS provides a more complete genomic view, enabling the improved detection of a broader spectrum of variants, including those in non-coding regions, intronic variants, short tandem repeats (STR), and copy-number variants (CNV) [13–15]. Recent publications evaluated genome sequencing’s ability to detect the most common diagnostic referrals in a large European hospital with detection rates of up to 95% in cases where these variants were already identified through conventional workflows [16, 17]. This makes GS a potentially very powerful unifying tool that could enhance diagnostic yield and reduce the time to diagnosis [18]. A recent review including 27 studies places the diagnostic yield of GS as a first-line test in rare disease at about 45% [19]. However, larger prospective studies focusing on the benefits of GS in a real-world setting are still scarce and do not evaluate all potential frameworks [19, 20]. In this clinically heterogeneous study, we aim to fill this gap by directly comparing the diagnostic yields of singleton genome sequencing (sGS), trio genome sequencing (tGS), and SoC to verify whether short-read GS can truly serve as a “one-test-for-all” strategy for rare disease cases. We focus on GS effectiveness as comprehensive diagnostic tools, capable of identifying a wide variety of genomic variants potentially missed by conventional SoC methods. Furthermore, our prospective and retrospective results highlight the feasibility of the three methods within the real-world setting of the second-largest university hospital in Germany [21]. We conducted a comparative analysis focusing on diagnostic yield, implementation processes, and overall practicality to determine which approach offers the optimal balance of diagnostic comprehensiveness and economic sustainability [22].

## Methods

### Cohort and study subjects

**Fig. 1:**
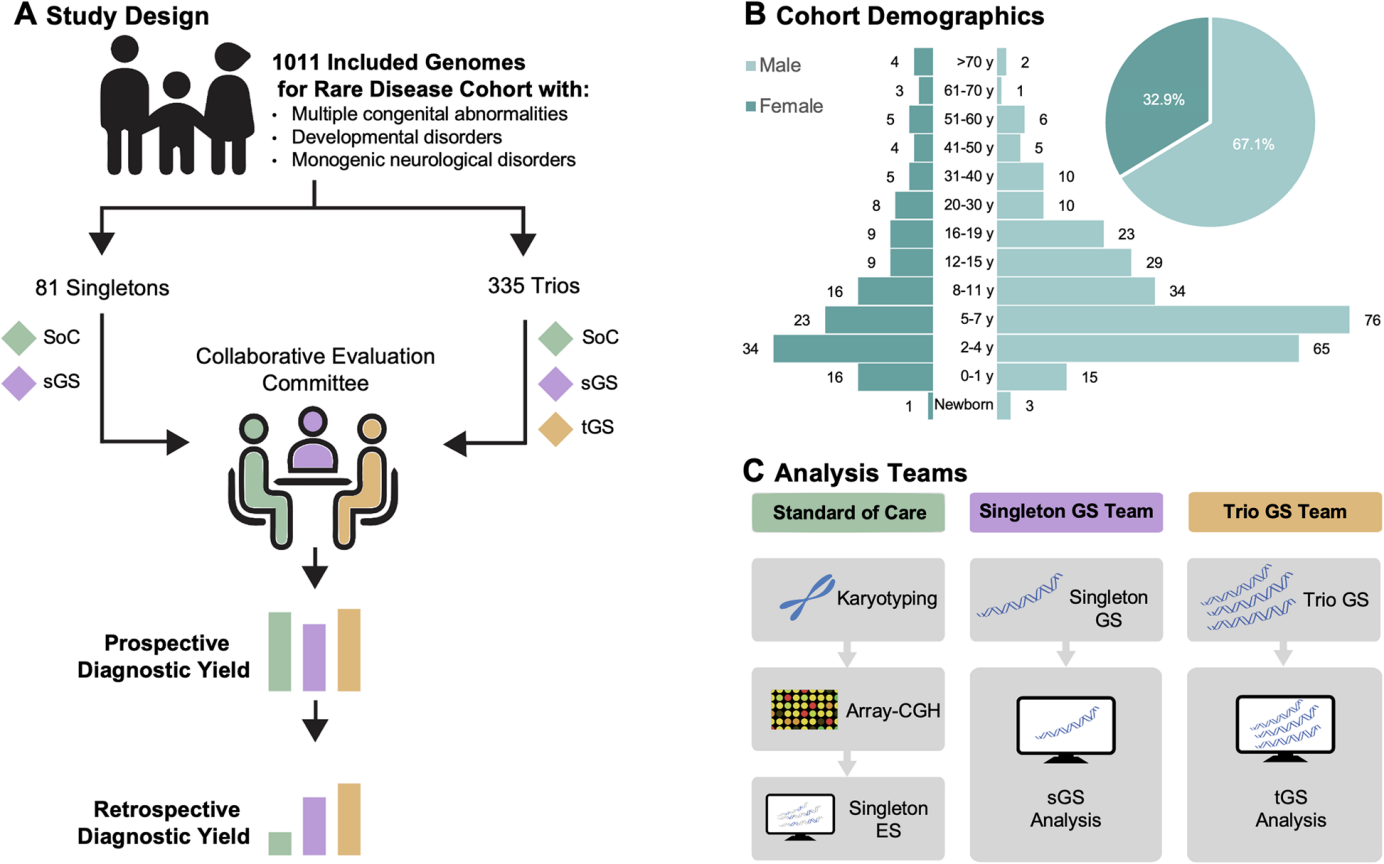
Study design and cohort overview for rare disease analysis. **A** In this study, our cohort comprised 1011 individuals from 416 index family cases of rare diseases. Among these, 81 singletons were analysed by the standard of care (SoC) and singleton genome sequencing (sGS) team, while 335 trio cases (multi-family member cases) received additional GS for family members and were further analysed by the trio genome sequencing (tGS) team. A collaborative evaluation committee assessed both prospective and retrospective results. **B** The demographic overview illustrates the study sex and age distribution of the 279 male and 137 female patients. **C** Three analysis strategies were employed: SoC (sequential testing of index blood samples with karyotyping, array-CGH, and exome sequencing), sGS (genome sequencing of the index patient), and tGS (genome sequencing of the index patient with parental and sibling data when available)

Between January 2022 and April 2023, the Universitätsklinikum Schleswig–Holstein (UKSH) Genome Consortium recruited patients from routine clinical care who met predefined study criteria and provided informed consent. This consent also covered the use of participants’ and relatives’ blood samples for the analysis with different strategies. Eligible participants included individuals with rare diseases such as developmental disorders, congenital abnormalities, syndromic conditions, or rare neurological syndromes. For all consenting participants, short-read GS was performed alongside SoC diagnostic strategies (Fig. 1A). Other inclusion criteria were a high likelihood of a genetic aetiology as determined by a clinical geneticist, no pre-existing clinical genetic diagnosis, no prior SoC or GS, eligibility to receive SoC covered by insurance, samples meeting technical quality control standards, and provision of full consent to participate in the study. Each index patient categorised with a syndromic or developmental disorder underwent SoC testing, including sequential analyses using karyotyping, array-CGH, and ES. If karyotyping or array-CGH identified a causal variant, ES was not performed. For other disease categories, such as neurological disorders, SoC included at least ES (see Table S3 for a case-wise overview). Additionally, GS was conducted on index patients and available family members, including parents and siblings. Ultimately, 1148 participants, including patients and their relatives, were recruited and sequenced, resulting in 448 index cases. Of the sequenced individuals, 416 index cases with 1011 genomes met inclusion criteria, while 32 cases were excluded. The cohort included 81 singletons, 51 duos, 258 trios, eleven quartets, a quintet, and a sextet. Ten quartet families and the sextet family involved two index patients, while the quintet family involved three. In the following, we will label all included singleton cases as singletons (81 cases) while all multi-family-member cases, regardless of size, will be grouped under trios (335 cases). Patients were categorised by disease groups to explore potential distinctions. Overall, 67.1% of the cohort were male and 84.8% of enrolled patients were below 20 years of age (Fig. 1B).

### Analysis strategies

In this prospective, blind study, three independent analysis teams analysed the cases (Fig. 1C):

- The SoC team analysed cases based on the phenotypic indications of the index patients’ karyotype, array-CGH, and ES data.
- The sGS team analysed cases based on the phenotypic indications and the index patients’ GS data.
- The tGS team focused only on trio cases and analysed them based on the phenotypic indications, the GS data of the index patients, and available siblings or parental samples.

Following their unique approach, each team committed to logging in only their candidate results without knowing the results of the other groups. The results were collected and assessed by a collaborative evaluation committee comprising representatives from each team, who determined the final diagnostic outcome reported to the patient.

Of the three independent teams that analysed the cases in this study, the SoC team comprised experienced members from the diagnostic department, highly proficient in routine analytical workflows such as karyotyping, array-CGH, and ES. The GS teams included analysts with only basic training in GS analysis, leading to comparatively less familiarity with the GS pipeline. Additionally, the GS pipeline itself was newly introduced and lacked the extensive validation and optimisation that the SoC pipeline had undergone.

Our three strategies were compared across two stages. The first stage, termed the prospective stage, involved evaluating prospective data submitted by each team. The prospective diagnostic yield refers to each team’s ability to identify the variants ultimately confirmed as the final diagnostic result by the committee.

The second stage, referred to as the retrospective stage, involved all teams evaluating the final results under strictly uniform conditions, assuming identical levels of expertise and experience across all teams. This stage aimed to determine whether the identified variants were technically detectable and, if so, whether they passed the analysis filters used in the different approaches (SoC, sGS, and tGS). By eliminating any influence of team-specific experience, the retrospective diagnostic yield purely reflects the inherent capability of each diagnostic strategy to identify the variants under fair and equal conditions.

### Genome sequencing and sample quality control

A total of 1148 DNA samples derived from patient blood underwent standard library preparation protocols using the DNA PCR-Free Prep, Tagmentation-Kit (Illumina, Inc., San Diego, CA, USA). This was followed by short-read GS with 16 samples per NovaSeq Flow Cell (Illumina, Inc.) on a NovaSeq™ 6000 instrument (Illumina, Inc.) using the NovaSeq 6000 S4 Reagent Kit (Illumina, Inc.) with 2 × 150 bp paired-end reads and an anticipated genome-wide coverage (mean mapped read depth) of 29-fold minimal; actual mean coverage of 38 × was documented. Due to technical issues or low sample quality, sequencing was repeated for 68 samples (6.55%). Adapting to a higher DNA concentration, transitioning from an initial 300 to 600 ng, substantially improved assay robustness.

### Exome sequencing and sample quality control

For ES, the coding regions of 19,433 genes were enriched using the Nextera Flex for Enrichment kit (Illumina, Inc.) and the xGen Exome Research Panel v2 (Integrated DNA Technologies, Inc., Coralville, IA, USA). Sequencing of the resulting libraries was conducted on the NovaSeq™ 6000 platform with 2 × 150 bp paired-end reads. Raw data were aligned to the human reference genome GRCh38 (hg38). The mean sequencing depth for index/mother/father samples was 202.8 ×/159.1 ×/155.2 ×, with 97.8%/97.0%/97.2% of target regions covered at least 20 × and 97.4%/96.3%/96.5% covered at least 30 ×.

### Genome and exome variant interpretation

Variant detection for genomes was performed using the Germline Pipeline of the DRAGEN™ (Dynamic Read Analysis for GENomics) Bio IT platform v3.7.5 (Illumina, Inc.),[23] Manta Structural Variant Caller (Illumina, Inc.) [24] for CNV detection and ExpansionHunter (Illumina, Inc.) [25] for detection of STRs. ES analysis was performed using the IKMB DRAGEN Pipeline (https://github.com/ikmb/dragen-variant-calling), based on the commercial DRAGEN Pipeline v3.10.4 (Illumina, Inc.) [26]. Variants with coverage below 20 × or with less than 20% variant reads were excluded.

GS data was interpreted using the TruSight Software Suite v2.6 (TSS, Illumina, Inc.)[27] while for ES data, variant analysis was performed using Alissa Interpret v5.4.2 (Agilent Technologies, Santa Clara, CA, USA) [28]. Variant calling was followed by the application of a series of filtering steps for variant prioritisation. For rare diseases, variants with allele frequencies below 1% for recessive inheritance and 0.1% for dominant inheritance based on the frequencies from the Genome Aggregation Database (gnomAD) [29] and the Exome Aggregation Consortium (ExAC) [29] were considered as well as known disease-causing variants from the literature.

Protein-altering variants and those affecting canonical splice sites (for ES: ± 10 bp) were assessed for functional relevance, conservation, and splicing impact using various in silico tools (e.g. CADD score) [30].

For genome-wide strategies, variants were evaluated across the entire genome. All candidate variants were reported if alignment matched with the analysed individual’s symptoms and inheritance pattern. Additionally, the tGS team tracked potentially promising de novo variants with unknown gene-disease mechanisms as scientific findings, prioritising them for future re-evaluation or submission to GeneMatcher databases [31]. All variants were associated with the patient’s phenotype using Human Phenotype Ontology (HPO)[32] terms and evaluated using databases such as OMIM,[33] PubMed, ClinVar,[34] Decipher,[35] and in silico prediction tools (e.g. CADD score) [30].

Allele frequencies were assessed using the Genome Aggregation Database (v3.1.2) [29]. Visual validation of variants was performed using the Integrated Genome Viewer (IGV; Version: 2.13.2–2.17.4) [36].

### Genome and exome variant classification

Variant classification followed the guidelines of the American College of Medical Genetics and Genomics (ACMG) and Association for Molecular Pathology (AMP) and the Association for Clinical Genomic Science (ACGS) [37, 38]. The diagnostic yield is categorised into three classifications:

1. P/LP: Pathogenic or likely pathogenic variants were identified as the main clinical indication that align with the patient’s phenotype and inheritance pattern.
2. VUS: Variants of uncertain significance were assessed as the main clinical indication that matched the patient’s phenotype and inheritance pattern, supported by a high level of evidence (“Hot VUS”).
3. No findings: No diagnostic variants were identified.

Moreover, the genomic data of the sequenced individuals (patients and relatives) was analysed to report for P/LP variants classified as secondary findings (SF) within actionable genes curated in the ACMG SF list v3.2 [39].

### Array-CGH and sample quality control

DNA was isolated from patient blood samples, with approximately 1 µg of DNA per sample. These samples were subjected to array-CGH using the Agilent SurePrint G3 Human CGH Microarray Kit (8 × 60 K) (Agilent, Inc.). Standard protocols for DNA labelling and hybridisation were followed, with scans performed on the Agilent DNA Microarray Scanner.

### Array-CGH data analysis

Array-CGH data was processed using Genomic Workbench software (v7.0) (Agilent, Inc.), which facilitated the detection of CNVs. CNV interpretation was performed by comparing identified variants to known genomic databases, including ClinGen [40], DECIPHER,[35] and OMIM,[33] with additional in silico analysis performed to assess potential pathogenicity. The results were cross-referenced with patients’ clinical phenotypes to determine relevance and potential clinical significance (variants classified as P/LP and strong VUS combined).

### Karyotype analysis

Metaphases of each case were G-banded with conventional trypsin-Giemsa staining and analysed at 400 or 550 band level according to referral reason [41]. Karyotypes were described following the guidelines of the International System for Human Cytogenetic Nomenclature (ISCN 2020) [42]. Digital image acquisition, processing, and evaluation were performed using NEON interface for case and image data software version 1.3 (MetaSystems, Altlussheim, Germany). Chromosomal abnormalities, such as aneuploidies, translocations, deletions, and duplications, were identified and classified. Findings were interpreted in the context of clinical phenotypes, with cross-referencing against established cytogenetic databases (e.g. DECIPHER,[31] OMIM[29]) to assess clinical significance.

### Statistical analysis

Diagnostic yield (case resolution) was treated as a binary outcome: cases with a pathogenic or likely pathogenic variant were coded as 1 (resolved), and cases with only a variant of uncertain significance (VUS) or no variant were coded as 0 (unresolved). For diagnostic yield calculations, cases with two pathogenic variants where only one was detected were assigned a value of 0.5; however, for binary statistical analyses, these intermediate cases were randomly split, coded as resolved (1) and as unresolved (0), to maintain the required binary structure. Paired proportions of resolved cases across the three strategies (SoC, sGS, tGS) were compared using Cochran’s *Q* test. Pairwise comparisons were performed using McNemar’s test with Bonferroni correction. Proportions of resolved cases were reported with 95% confidence intervals (CI, Wilson score method). Analyses were conducted separately for prospectively and retrospectively collected data: the prospective analysis reflects team learning effects over time, whereas the retrospective analysis accounts for technical detection limits, isolating strategy-specific performance. The primary statistical hypothesis tested whether trio genome sequencing (tGS) provides a clinically meaningful improvement in diagnostic yield compared to standard-of-care (SoC) testing, defined as an absolute difference of ≥ 3 percentage points. All tests were two-sided; *p* values > 0.05 were not interpreted as evidence of a difference. Statistical analyses were carried out in R (version 4.3.1).

### Comparison and learning curve

Case comparisons between teams began after the first 100 cases, with results subsequently shared among the groups. This approach enabled continuous error identification and inferred adjustments to minimise disparities stemming from potential experience gaps within the teams. To further dissect the nature of missed diagnoses, all variants that were missed by any team were tracked and categorised according to whether the missed variants were attributable to experience-related factors or to technical limitations. To illustrate this process, the prospective diagnostic yield was tracked using a sliding window analysis, capturing temporal changes in performance and optimisation (Table S3). A window size of 25 cases before and after each case was selected to calculate the average diagnostic yield for each team based on the total number of cases analysed. These learning and adaptation curves, displayed with linear regression, were used to monitor trends and evaluate the performance of each strategy across all 335 trio cases.

### Language editing and AI-assisted copy editing

ChatGPT (Version GPT-4o, OpenAI) [43] was used exclusively for copy editing to improve readability and language. The authors reviewed and refined all content, assuming full responsibility for the final manuscript.

## Results

### Diagnostic yield

Between January 2022 and April 2023, 448 cases with 1148 patients and their relatives were recruited, sequenced, and analysed. After quality control, 1011 genomes from 416 cases were retained for analysis, while 32 cases were excluded, yielding a cohort of 335 trios and 81 singletons (Fig. 1A). The cohort was predominantly male (67.1%), and most patients (84.8%) were younger than 20 years (Fig. 1B).

**Fig. 2:**
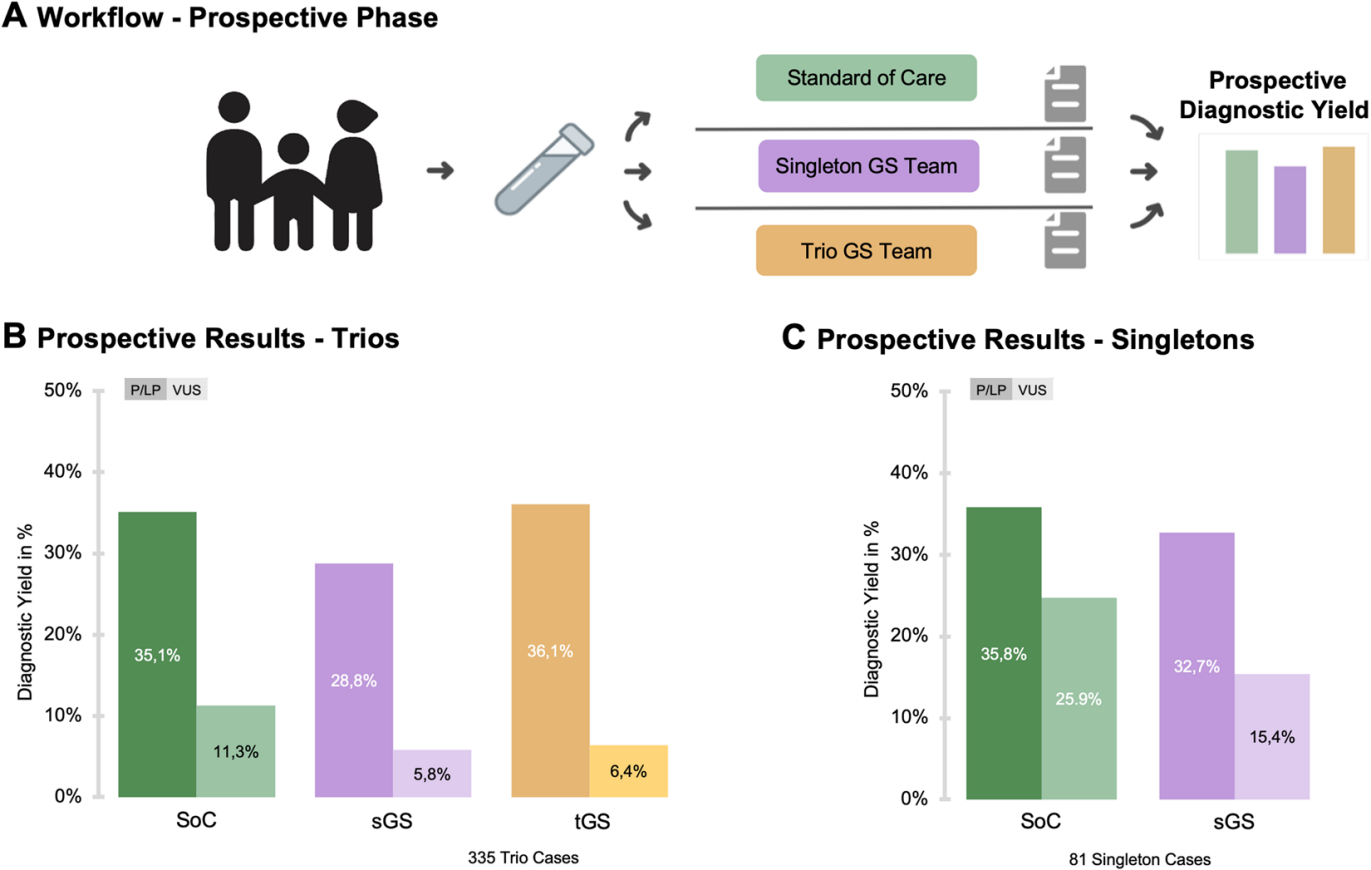
Prospective workflow and diagnostic yield across three analysis strategies. **A** Workflow for the analysis of prospective data with evaluation by the collaborative evaluation committee. **B** Prospective diagnostic yield for 335 trio cases, comparing the standard of care (SoC), singleton genome sequencing (sGS), and trio genome sequencing (tGS) strategies, with a focus on pathogenic or likely pathogenic (P/LP) variants and variants of uncertain significance (VUS). **C** Prospective diagnostic yield for 81 singleton cases, comparing the SoC and sGS strategies, focusing on P/LP variants and VUS

The initial evaluation of the 335 trio cases revealed the following diagnostic yields for P/LP variants: 35.1% (*n* = 117.5 cases) for SoC, 28.8% (*n* = 96.5 cases) for sGS, and 36.1% (*n* = 121 cases) for tGS. When including VUS, the combined percentage of cases with clinically significant variants increased to 46.4% (*n* = 155.5 cases) for SoC, 34.6% (*n* = 116 cases) for sGS, and 42.5% (*n* = 142.5 cases) for tGS. For the evaluation of 81 singleton cases, the differences between SoC and sGS were smaller. SoC identified P/LP variants in 35.8% of cases (*n* = 29) and VUS in 25.9% of cases (*n* = 21), yielding a total diagnostic rate of 61.7%. In comparison, sGS detected P/LP variants in 32.7% of cases (*n* = 26.5) and VUS in 15.4% (*n* = 12.5 cases), for a total diagnostic yield of 48.1% (Fig. 2).

**Fig. 3:**
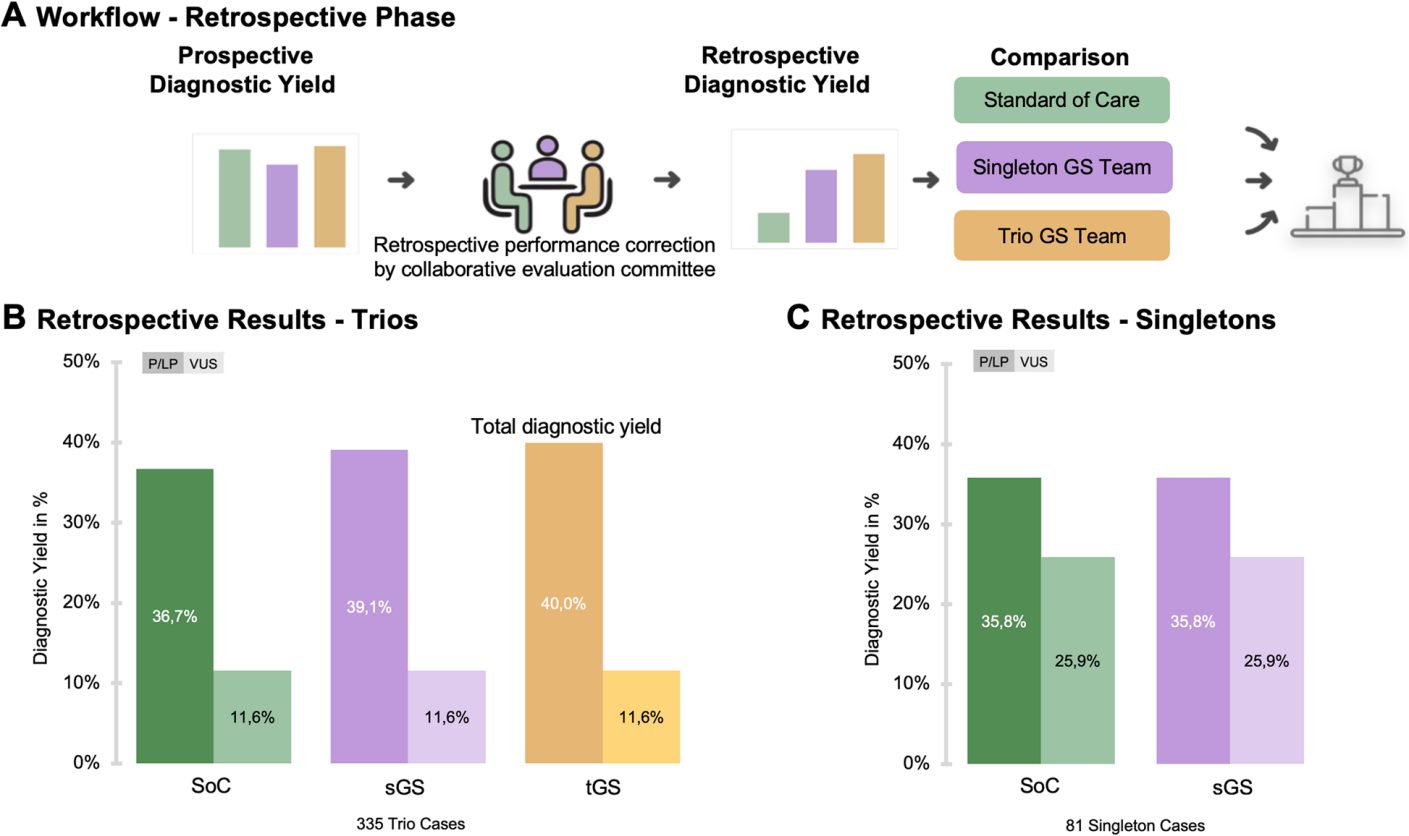
Retrospective diagnostic yield. **A** Workflow for the retrospective analysis of prospective data following evaluation by the collaborative evaluation committee. **B** Retrospective diagnostic yield for 335 trio cases, comparing the standard of care (SoC), singleton genome sequencing (sGS), and trio genome sequencing (tGS) strategies, with a focus on pathogenic or likely pathogenic (P/LP) variants and variants of uncertain significance (VUS). The tGS covered all identified variants (total diagnostic yield). **C** Retrospective diagnostic yield for 81 singleton cases, comparing the SoC and sGS strategies, focusing on P/LP variants and VUS

The prospective evaluation stage was followed by a retrospective assessment of results to address experience gaps in evaluating the strategies. The curated data for all 335 trio cases revealed a retrospective diagnostic yield for P/LP variants of 36.6% (*n* = 123 cases) for SoC, 39.1% (*n* = 131 cases) for sGS, and 40.0% (*n* = 134 cases) for tGS. The percentage of VUS was consistent across all strategies at 11.6% (*n* = 39 cases). Including the VUS category, the combined percentage of cases with a variant with clinical significance was 49.6% (*n* = 145 cases) for SoC, 52.3% (*n* = 154 cases) for sGS, and 53.2% (*n* = 157 cases) for tGS. In the retrospective analysis of the 81 singleton cases, SoC and sGS demonstrated identical performance, with 35.8% (*n* = 29) for P/LP variants and 25.9% (*n* = 21 cases) for VUS. For these cases, no variants were exclusive to either approach (Fig. 3).

### Statistical evaluation of diagnostic yields within trio cases

The statistical analysis using Cochran *Q* test of the prospective data revealed significant differences in diagnostic yield across sequencing strategies (*p* < 5.35 × 10^−5^) for the 335 trio cases. The sGS strategy showed the lowest diagnostic yield for P/LP variants (28.7%, 95% CI: 23.8–33.5%), whereas SoC (34.9%, 95% CI: 29.8–40.0%) and tGS (36.1%, 95% CI: 31.0–41.3%) had comparable yields. Pairwise comparisons confirmed sGS underperformed relative to both SoC (*p* = 0.009, Δ*p* = 6.2%) and tGS (*p* = 0.001, Δ*p* = 7.4%), whereas SoC and tGS showed no difference (*p* = 0.203, Δ*p* = 1.2%).

Statistical analysis of the retrospective data revealed differences across strategies (*p* = 0.000148), with tGS achieving the highest diagnostic yield (40.0%; CI: 34.8–45.2%), followed closely by sGS (39.1%; CI: 33.9–44.3%) and SoC (36.7%; CI: 31.6–41.9%). While sGS and tGS were similar (*p* = 0.745, Δ*p* = 0.9%), both outperformed SoC (sGS vs. SoC: *p* = 0.04, Δ*p* = 2.4%; tGS vs. SoC *p* = 0.008, Δ*p* = 3.3%). Our results support the primary hypothesis: in the retrospective comparison, tGS achieved a clinically meaningful 3.3% absolute improvement in diagnostic yield over SoC, meeting our predefined threshold and underscoring its utility in accelerating diagnoses, particularly through the detection of unique variant types identifiable only by GS strategies.

### GS unique variants

**Fig. 4:**
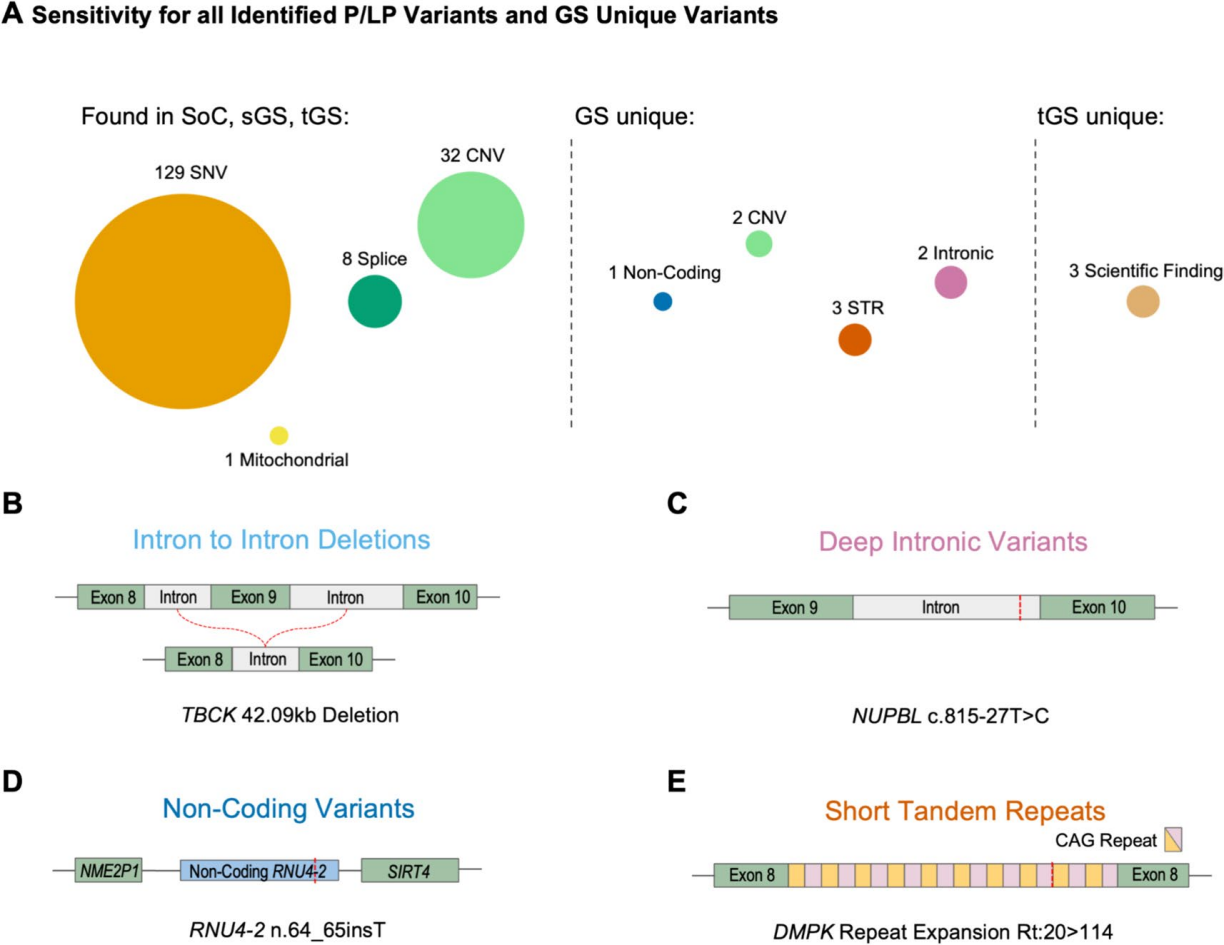
Strategy performance for variant types and unique genome variants. **A** The sensitivity for detecting specific variant types varied across the tested strategies. Of the 181 pathogenic or likely pathogenic (P/LP) variants identified within the cohort, 170 were detected using the standard of care (SoC), singleton genome sequencing (sGS), and trio genome sequencing (tGS) strategies, encompassing single-nucleotide variants (SNVs), copy-number variants (CNVs), splice variants, and mitochondrial variants. Unique variant types identified through GS included additional intron-to-intron CNVs, deep intronic variants, non-coding variants, and short tandem repeats (STRs). Notably, tGS identified three additional de novo variants, later reclassified as P/LP based on GeneMatcher collaborations and emerging studies linking new gene-disease associations. **B**–**E** Representative examples include an intron-to-intron deletion (CNV), a deep intronic variant, a non-coding variant, and an STR repeat tract expansion (Rt) detected using GS strategies

A total of 223 cases, singletons and trios combined, were identified with a diagnostic genetic cause (P/LP and VUS) based on the final retrospective results. According to ACMG/AMP criteria, 163 cases had variants classified as P/LP, while 60 cases were classified with VUS. In total, 181 disease-causing variants were identified (Table S1), 170 of which were detectable using the SoC approach, with 11 additional variants exclusively identified through the GS pipeline (Fig. 4A).

The variants covered by all strategies included 32 CNVs, 129 coding SNVs, 8 splice variants, and one mitochondrial variant. Among the GS unique variants, we detected eight variants in total by sGS and tGS: two intragenic CNVs spanning from one intron to another intron, three STRs, two intronic variants, and one non-coding variant (Table S4a). Unique to the tGS strategy, we discovered three de novo variants that initially lacked a gene-disease association but could later be upgraded to LP via either a GeneMatcher cooperation with similar patients or a subsequent publication (Table S4a) [44]. GS identified STRs in three patients: two with estimated CAG repeat expansions of 143 and 114 in the *DMPK* gene, and one with a CGG repeat expansion of 90 in the *FMR1* gene. Here, genome sequencing (GS) served as the primary screening method. The *FMR1* variant initially identified as a premutation by GS was subsequently confirmed as a full mutation with over 200 CGG repeats through orthogonal targeted Fragile X testing (Fig. 4B–E). These screening capabilities were similarly demonstrated in the singleton case #249, with two variants in *RIPK4* that were later reclassified as likely pathogenic based on additional evidence from a phasing assay, which revealed that the variants were positioned in trans. Additionally, four variants classified as VUS were identified as strong candidates for further functional validation: #181 (*ARID1B*), #35 (*KAT6B*), and #296 (*KMT2D*) based on their association with specific methylation episignatures, and #207 (*CACNA1D*), a splicing variant with predicted RNA-level effects that could support reclassification using RNA sequencing.

During the study, publications on recently discovered pathogenic variants within the non-coding snRNA *RNU4-2* locus highlighted the potential of GS strategies for reanalysis [45]. We focused on the recently uncovered disease potential of snRNAs from the *RNU* family. A comprehensive VCF reanalysis of the full cohort was conducted, targeting specifically genomic regions for *RNU4-2*, *RNU2-2*P, *RNU5A-1*, and *RNU5B-*1. This approach enabled the mapping and classification of potential variants. Notably, we identified a patient carrying the most common previously described disease-causing variant in the *RNU4-2* critical region, n.64-65insT, which is associated with impaired RNA splicing and contributes to the patient’s neurological symptoms (Fig. S2) [46].

### Patterns of inheritance and variant types

**Fig. 5:**
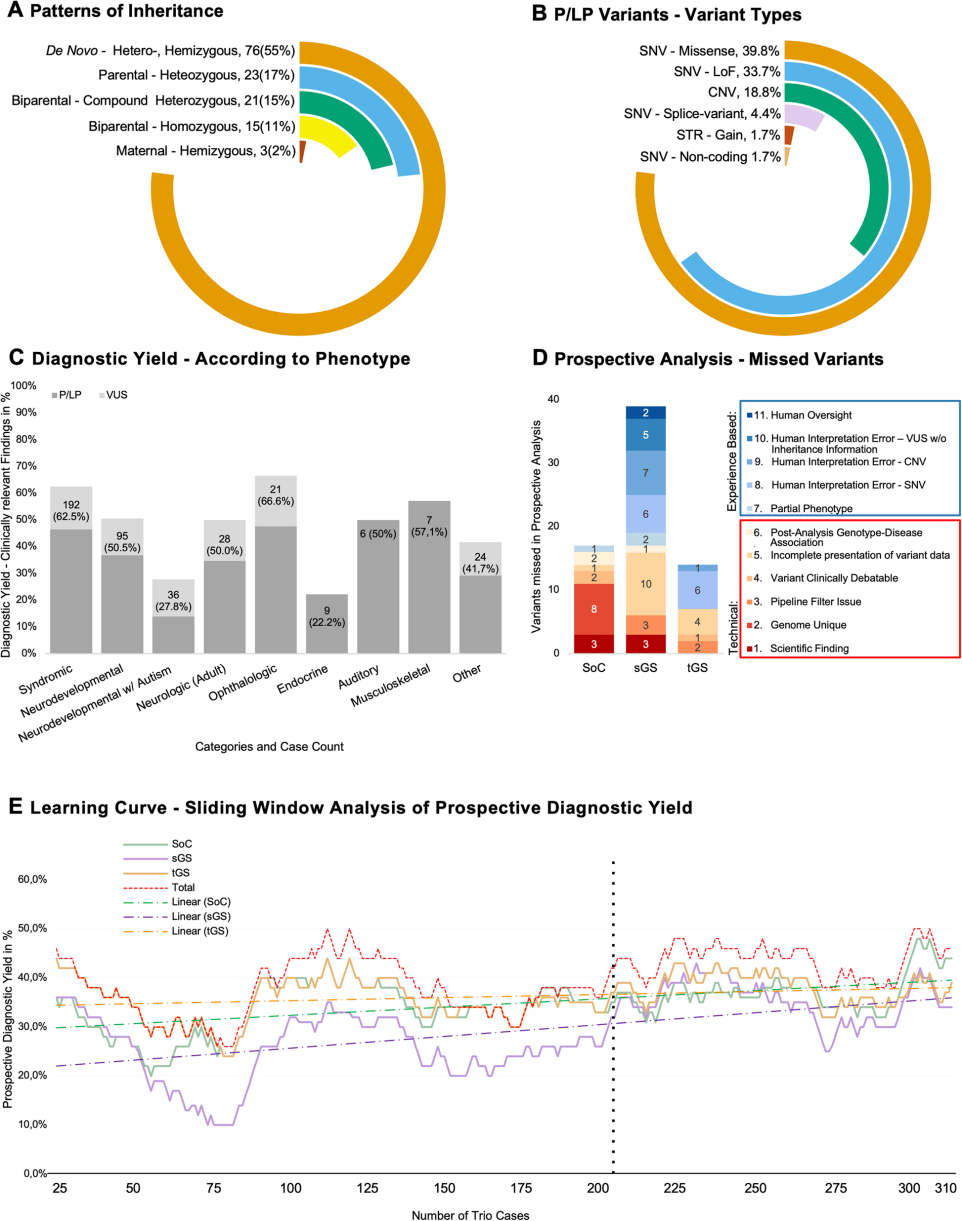
Inheritance and variant types/disease categories/learning curve. **A** The distribution of inheritance patterns for genetically identified diseases solved with pathogenic or likely pathogenic (P/LP) variants. **B** All 181 P/LP variants are classified by type, including missense small nucleotide variants (SNVs), loss-of-function (LoF) SNVs, splice-effect SNVs, non-coding SNVs, short tandem repeats (STRs), and copy-number variants (CNVs). **C** Retrospective diagnostic yield is presented for various disease categories within the cohort, categorised based on the final diagnostic outcome. **D** Bar chart showing the proportion of missed variants categorised as either technical or experience-related across the three diagnostic strategies. Each bar represents one strategy and is subdivided into two categories based on root cause classification: technical (categories 1–6) and experience-related (categories 7–11). **E** A sliding window analysis (25 cases before and after each case) evaluates the prospective diagnostic yield for trio cases with P/LP variants. The comparison includes standard of care (SoC), singleton genome sequencing (sGS), and trio genome sequencing (tGS), with a dotted line at 205 cases representing a symbolic break-even point for all strategies, defined as a 1% diagnostic yield difference

From the 115 trio cases in which we identified P/LP variants responsible for the individuals’ phenotypes and both parental genomes were available, we assessed 138 unique variants and their patterns of disease inheritance. Here, 55.1% of the inheritance patterns were de novo, 17.7% maternally or paternally inherited heterozygous, 15.2% were biparental compound heterozygous, 10.9% biparental homozygous, and 2.2% maternal hemizygous (Fig. 5A).

Among the 181 P/LP variants identified, single-nucleotide variants (SNVs) were the most common, comprising 39.8% missense variants, 33.7% loss-of-function (stop gain, start loss, frameshift), 18.8% CNVs, 4.4% splice-region SNVs, and 1.7% intronic or non-coding SNVs. STRs also represented 1.7% (Fig. 5B).

### Disease categories and their respective diagnostic yields

Syndromic (*n* = 192), neurodevelopmental (*n* = 95 + 36 = 131), and neurological (*n* = 26) cases represented the largest proportion within our cohort. The retrospective diagnostic yield for clinically significant findings in syndromic cases was 62.5%. However, this yield varied significantly across different disease categories, highlighting differences in diagnostic outcomes. This was particularly evident in the 36 cases of neurodevelopmental disorders involving autism, which had a combined diagnostic yield of 27.8% for clinically significant variants, compared to the 95 cases of neurodevelopmental disorders without autism, which demonstrated a yield of 50.5% (Fig. 5C).

### Secondary findings

In our cohort, SF in genes included in the ACMG SF v3.2 list [35] for reporting SF in clinical exome and genome sequencing were identified in 2.4% of the index patients within all 416 cases (Table S5). In contrast, the prevalence of identified SF in parents and sequenced siblings was only observed at 1.8% in the 335 trio cases (595 samples).

### Prospective implementation of GS in the clinic

During the study, the tGS team reported 255 candidate variants across 335 trio cases, compared to 312 reported by the SoC team and 414 by the sGS team. In the 81 singleton cases, 69 candidate variants were identified by the SoC team, while 110 were reported by the sGS team, highlighting variability in candidate reporting between teams with differing levels of expertise, access to analytic data supporting variant identification, and adaptation to analytical methods.

To address heterogeneity in team experience and monitor the optimisation process, the diagnostic yield of the three approaches was continuously evaluated and openly reviewed by a collective evaluation committee beginning after the first 100 cases. A sliding window visualisation revealed that after 74 cases, the diagnostic yield was 10% for the sGS team, compared to 28% for the tGS team and 26% for the SoC team. Despite this initial disparity, the sGS team exhibited the steepest improvement, eventually matching the performance of the other teams after 205 cases (within a 1% diagnostic yield deviation) and briefly surpassing them after 229 cases, likely due to the steadily increasing experience and enhanced workflow efficiency, reflecting a successful adaptation to the initial gaps in experience and pipeline integration. The tGS approach, enhanced by the inclusion of additional inheritance data for candidate variants, demonstrated the highest yield closely followed by the SoC framework (Fig. 5E). To investigate why certain diagnoses were missed, all false negatives were classified as either experience-related or technical (Table S7). On the technical side, the most significant cause of missed variants in the genome-based strategies was the incomplete presentation of variant data, where the pipeline lacked access to or inconsistently processed the most up-to-date variant information. In contrast, the technical errors in the SoC were primarily due to its inherent limitations (genome-specific category). In the two GS-based strategies, which were primarily executed by less experienced personnel, approximately 50% of missed variants were attributable to experience-related factors. In contrast, the SoC team, staffed with experienced diagnosticians, showed just one experience-related miss; all other missed variants in this group were due to technical limitations (Fig. 5D).

#### Effects of inheritance

The ACMG/AMP criteria PS2 (confirmed de novo variant with established parental identity) and PM6 (assumed *de novo* without confirmed maternity/paternity) are pivotal for interpreting dominant variants, with PS2 considered stronger due to its reliance on robust parentage confirmation, often achievable via Sanger sequencing combined with microsatellite analysis or in our case having access to the tGS data.

In the trio cohort where the inheritance of a variant was confidently identified for 115 cases (138 variants), 15 cases with 15 unique variants would be downgraded to VUS in the absence of ACMG criterion PS2. Additionally, in four duo and 21 singleton cases, we identified 28 VUS variants (24 unique VUS for singleton and four unique VUS for the duo cases) that could have been hypothetically upgraded to LP/P through application of ACMG criteria PS2 or PM6. Notably, in the singleton cohort, 33.3% (8 variants) of these 24 unique VUS strictly required the more robust PS2 criterion for potential reclassification. Cumulatively, for the singleton cohort, all 24 VUS represented candidates for potential upgrade through added inheritance data (Table S6). This highlights the potential strength of inheritance data in classification of potentially disease-causing variants.

## Discussion

In this study, we compared short-read GS (sGS and tGS) to SoC methods for diagnosing rare diseases. The tGS team had the highest diagnostic yield with P/LP variants in 40.0% of cases, followed by sGS at 39.1%, and both outperforming SoC at 36.7%. GS’s broader coverage and inheritance data improved the identification of complex structural and non-coding variants. Additionally, tGS uncovered de novo variants that were initially considered candidate causal variants and were subsequently validated as disease-causing through GeneMatcher collaborations or literature review. These results align with Tan et al.’s comparison of singleton and trio ES, which demonstrated improved detection of causal de novo variants without established gene-disease associations. Notably, their study involved a relatively small cohort of 30 rare disease cases, demonstrating a 3.3% increase in diagnostic yield with the trio approach [47].

Previous studies have suggested that GS has the potential to serve as a universal diagnostic tool for rare diseases. Our data confirm this for tGS in this prospective real-world study, aligning well with the findings of Schobers et al. that GS effectively functions as a potential “one-test-for-all” strategy, covering all variants reported by our SoC workflow [16]. This referenced study reported that short-read GS detected 94.9% of 1271 clinically relevant variants previously detected by alternative established diagnostic methods in 1000 cases, covering small, large, and structural variant types. Overall, tGS demonstrated consistent superiority over SoC in both our datasets, with a clinically meaningful 3.3% improvement in diagnostic yield in the retrospective comparison.

A notable secondary benefit of tGS is its ability to identify SF in ACMG-designated actionable genes in both the index patient and the parents. These findings can guide preventive care and early interventions, such as cancer screenings or lipid-lowering therapies, thereby reducing morbidity and mortality. In this study, 2.4% of index patients and 1.8% of trio-case parents and siblings had actionable SF. For relatives, this is slightly lower than anticipated based on prior studies [48].

On the other hand, retrospective parity between sGS and tGS, despite tGS’s numerical superiority, positions sGS as a viable alternative when parental samples are unavailable, particularly in resource-constrained settings. However, short-read GS approaches face limitations, particularly in phasing haplotypes, resolve complex structural variants (especially balanced events), mapping complex genomic regions of high homology such as repetitive sequences, segmental duplications, or specifically long STRs [16]. For our identified STR-expansions, short-read GS could only detect potential expansions in repetitive motifs, emphasising this key limitation of GS as a standalone diagnostic tool. This was also the case for two unbalanced translocations, where GS identified the clinically relevant copy number change but lacked the information to characterise the event as a translocation. More broadly, although short‐read GS enables comprehensive variant detection, its interpretive power can be limited in the absence of complementary data, particularly when variants are located in low-complexity regions. Secondary analysis like DNA methylation analysis and multiomics can help clarify VUS, while long-read GS is needed to precisely measure motif length and fully characterise complex genomic variation. Nonetheless, GS remains a valuable first-tier screening method, guiding the targeted selection of cases for deeper multiomic investigation to resolve ambiguous findings.

An increasing challenge in cases with inconclusive findings is the growing demand for the reanalysis of genomic data. In particular, automated processes have been proposed as a means to streamline and enhance the reanalysis of patient genomic data [49]. A recent meta-analysis of 29 studies has shown that reanalysing ES and GS data starting approximately 2 years or later after the initial analysis can provide conclusive findings for about 10% of previously inconclusive cases [50]. This study underscores the key advantage of GS as a potential alternative to SoC, partly because of its superior reanalysis capabilities. The comprehensive coverage offered by GS enables reexamination without the need for additional resampling or resequencing, enhancing diagnostic performance, especially for non-coding regions and areas that are poorly understood [51, 52]. Within this ongoing study, employing adaptive reanalysis of newly identified disease-relevant non-protein-coding regions, such as the *RNU4-2* gene locus, led to the resolution of a previously undiagnosed case [45, 46].

Our results highlight the successful integration of GS into a diagnostic framework traditionally dominated by a series of elaborate methods. The implementation in this prospective study required an extensive training and workflow optimisation period for the less experienced genomic teams, addressing challenges posed by the comprehensive data interpretation. Initial discrepancies in expertise between SoC and sGS visible in our learning curve underscored the need for adaptation to the new approaches, better filtering techniques, and platform improvements. Remarkably, for tGS the provided inheritance data helped to heavily offset experience gaps to support a more accurate interpretation. Thus, we demonstrated that the primary advantage of tGS is its capacity to allow even less experienced teams to achieve diagnostic yields on par with those of experienced SoC teams. Ultimately, through our prospective approach, including a continuous iterative performance comparison, we were able to bridge the experience gap and improve on technical hurdles in real-time, refine workflows, and enhance sGS capabilities, thereby achieving diagnostic yields more comparable to retrospective analyses. This shows that sGS could potentially be a cost-effective alternative to tGS in a clinical environment with high expertise levels.

Still, sGS conducted without parental data resulted in a higher proportion of VUS and necessitated the evaluation of more candidate variants due to the inability to exclude them based on segregation. The inheritance data enable stronger classification of putative de novo variants through the ACMG criterion PS2, thereby improving the detection of pathogenic variants. In sGS, the absence of PS2 and PM6 explains the higher proportion of reported VUS. Even if segregation were retrospectively performed in sGS, the lack of confirmed maternity and paternity would restrict classification to the weaker ACMG criterion PM6. Consequently, despite demonstrating a de novo mechanism, this limitation would perpetuate an elevated number of VUS.

Consequently, trio analysis required the fewest variants to be reported while simultaneously achieving the highest diagnostic yield, demonstrating both time efficiency and clinical effectiveness.

In singleton cases, the observed difference in prospective diagnostic yield between standard-of-care (SoC) and singleton genome sequencing (sGS) was less pronounced than in trio analyses. This discrepancy, noted earlier, likely reflects underreporting of variants of uncertain significance (VUS) by a less experienced team, where limited interpretability hindered definitive classification. Notably, several of these unreported VUS may have been reclassified as (likely) pathogenic if segregation data had been available. This persistent diagnostic gap between sGS and SoC in the singleton subcohort underscores the importance of expert VUS interpretation, particularly in the absence of parental data.

Of the genetically identified diseases caused by P/LP variants, 15.8% followed autosomal dominant inheritance from a parent, exceeding expectations. This higher rate reflects not only known familial conditions or reduced penetrance but also incomplete parental phenotypic descriptions. In contrast to the anticipated 64% predominance of de novo variants, these findings highlight the complexity of inheritance patterns and the need for thorough genetic anamnesis.

The utility of diagnostic solutions depends on both cost and time efficiency. GS’s “one-test-for-all” approach significantly reduces analysis time compared to the sequential SoC methods [19, 53]. A recent study demonstrated that rapid tGS could diagnose rare diseases in 290 critically ill infants in under 3 days [47]. In our study, GS facilitated the identification of disease-causing variants, enabling faster clinical decisions and improving diagnostic yield. However, a direct comparison of our time-to-diagnosis metrics was scientifically infeasible due to differences in study design, analytical strategies, and the varying levels of training and adaptation across the three teams. Notably, tGS eliminated the need for additional segregation analysis, showing a benefit in time even without a formal time-to-diagnosis analysis, making it especially valuable for time-sensitive contexts such as paediatric or acute care, where rapid diagnosis is paramount.

## Conclusions

In summary, our study demonstrates the advantages of integrating short-read GS into rare disease diagnostics, challenging long-held assumptions about conventional diagnostic approaches. Our work is unique in several respects. First, to our knowledge, it provides the first prospective, direct, head-to-head comparison of sGS trio versus tGS in a real-world clinical setting. Second, it offers a time-resolved analysis of diagnostic learning curves, showing how diagnostic yield improves with increasing analytical experience. Third, by systematically reviewing missed cases, the study identifies recurring sources of error, providing valuable insights for other laboratories to refine training strategies and adapt their pipelines. By linking diagnostic outcomes to both analytical design and user expertise, we offer a practical framework for implementing genome sequencing in routine diagnostics. Our findings could have a direct impact on diagnostic and academic laboratories worldwide, especially where cost constraints remain a key consideration in genetic testing. tGS showed the highest diagnostic yield, identifying 40% of P/LP variants and maintaining robust performance even with less experienced teams. sGS, while slightly less comprehensive, remains a cost-effective alternative that achieves comparable diagnostic success when performed by experienced teams. Both approaches demonstrate the potential to increase the detection of clinically significant variants and to match or surpass SoC performance.

## Supplementary Information

Additional file 1: Supplementary tables. Table S1 Variant and case-level summary across the cohort. Table S2 All variants classified by ACMG criteria. Table S3 Prospective and retrospective diagnostic yield. Table S4a + b Genome unique variants and scientific de novo findings. Table S5 Secondary findings based on ACMG SF v3.2 list. Table S6 Variants with ACMG classification that can be directly affected by their access to inheritance information. Table S7 P/LP variants missed in the prospective analysis by each team and corresponding reasons.

Additional file 2: Supplementary figures. Fig. S1 Variant classification framework for cases with a molecular diagnosis. Fig. S2 *RNU4-2* reanalysis.

## Data Availability

Summary-level variant data have been included in the supplementary materials. All clinically relevant variants identified in this study have been submitted to ClinVar [54]. Raw sequencing data are not publicly available due to ethical and legal restrictions but may be available upon request with appropriate approvals. Data can be made available upon reasonable request to the corresponding author. If approved, the data will be provided within a preparation period of up to four weeks.

## Abbreviations

GS: Genome sequencing
sGS: Singleton genome sequencing
tGS: Trio genome sequencing
SoC: Standard of care
array-CGH: Array comparative genomic hybridisation
ES: Exome sequencing
STR: Short tandem repeat
CNV: Copy-number variant
VUS: Variant of uncertain significance
P/LP: Pathogenic/likely pathogenic
ACMG: American College of Medical Genetics and Genomics
AMP: Association for Molecular Pathology
ACGS: Association for Clinical Genomic Science
SF: Secondary finding
CI: Confidence interval
