# Supplementary material for: Evaluating genome sequencing strategies: trio, singleton, and standard testing in rare disease diagnosis
Source: Genome Med. 2025 Sep 18;17:100. doi: 10.1186/s13073-025-01516-7 (PMC12445032; doi:10.1186/s13073-025-01516-7)
Supplement: Supplementary file 2 — Additional file 2: Supplementary figures. Fig. S1 Variant classification framework for cases with a molecular diagnosis. Fig. S2 RNU4-2 reanalysis. [file 13073_2025_1516_MOESM2_ESM.pdf]

Additional file 2: Supplementary Figures

Fig. S1: Variant Classification Framework for Cases with a Molecular Diagnosis

Fig. S2: *RNU4-2* Reanalysis

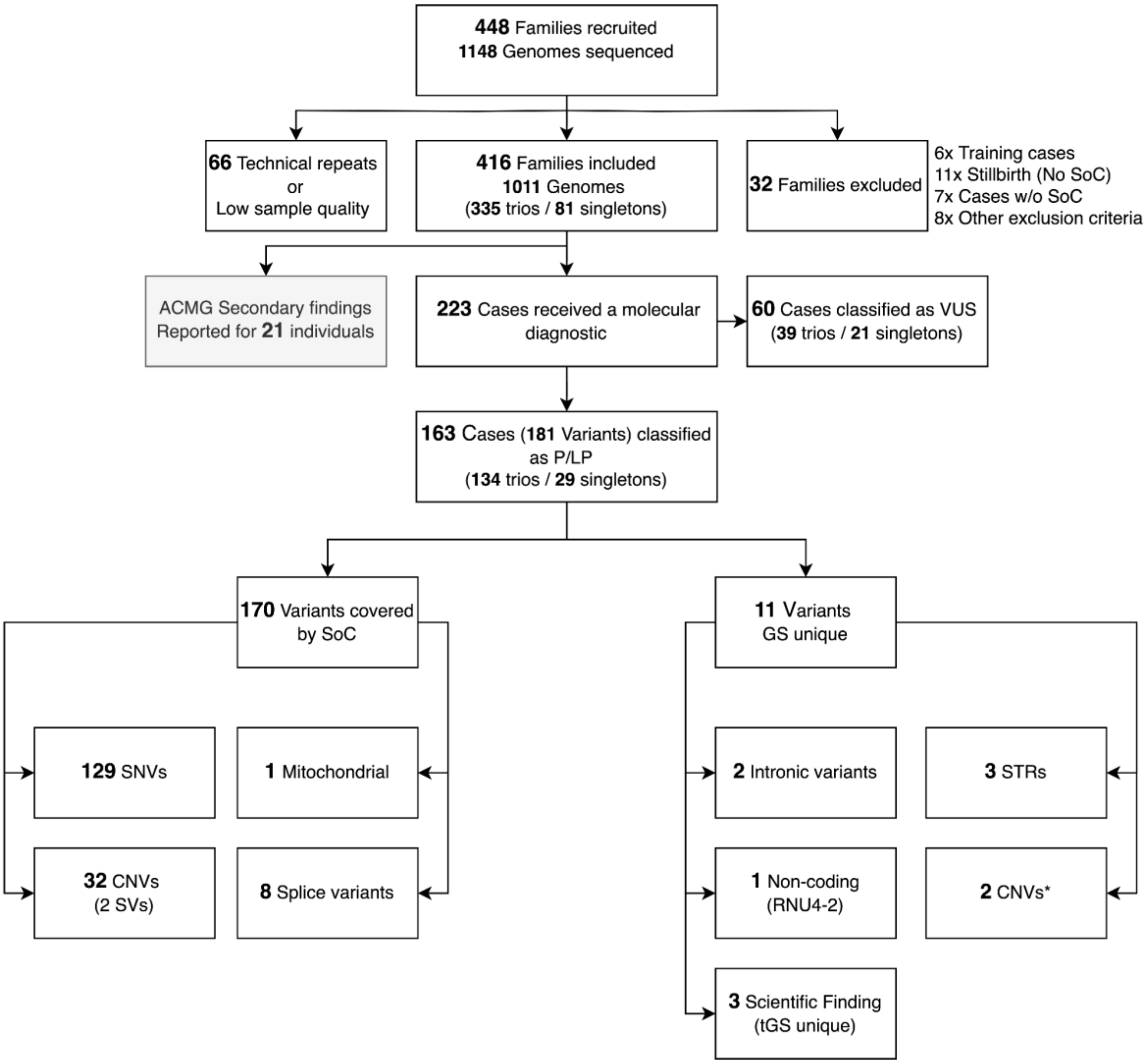

Supplementary Fig. S1 Variant Classification Framework for Cases with a Molecular Diagnosis

Initial recruitment selected 448 cases for sequencing; 32 cases were ultimately excluded. Among the 416

included index cases diagnostic variants were identified in 223 cases. Of these, 163 cases had final diagnostic results with variants classified as pathogenic or likely pathogenic (P/LP), while 60 cases involved variants of uncertain significance (VUS). In total, 181 P/LP variants were identified; 170 were detectable by standard-of-care (SoC) methods, including 32 copy-number variants (CNVs), 129 single-nucleotide variants (SNVs), eight splicing variants, and one mitochondrial variant. An additional eleven variants were identified exclusively through genome sequencing (GS), including two intron-to-intron CNVs, three short tandem repeats (STRs), two deep intronic variants, one non-coding variant, and three novel *de novo* variants. The latter were identified through GeneMatcher associations made possible by trio genome sequencing (tGS). For 21 individuals we identified an ACMG secondary finding (SF) variant. *Likely detectable by SoC (ES) with improved CNV calling.

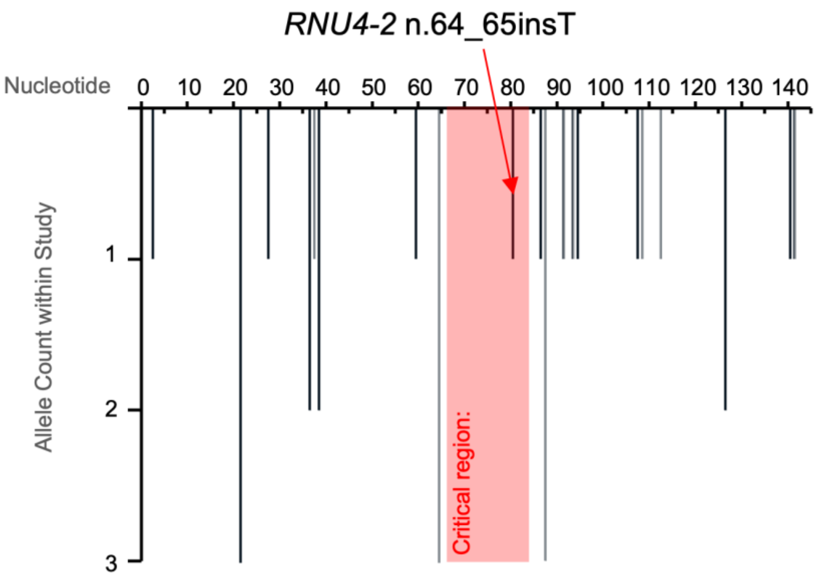

Supplementary Fig. S2 RNU4-2 Reanalysis

The graphic displays allele counts for all variants identified within our cohort in the *RNU4-2* locus, including our reported pathogenic variant, located within the 18 bp critical region.
